# The Effect of Hyperparathyroid State on Platelet Functions and Bone Loss

**DOI:** 10.4274/tjh.2015.0087

**Published:** 2016-12-01

**Authors:** Göknur Yorulmaz, Aysen Akalın, Olga Meltem Akay, Garip Şahin, Cengiz Bal

**Affiliations:** 1 Eskişehir State Hospital, Clinic of Endocrinology, Eskişehir, Turkey; 2 Eskişehir Osmangazi University Faculty of Medicine, Department of Endocrinology, Eskişehir, Turkey; 3 Eskişehir Osmangazi University Faculty of Medicine, Department of Hematology, Eskişehir, Turkey; 4 Eskişehir Osmangazi University Faculty of Medicine, Department of Nephrology, Eskişehir, Turkey; 5 Eskişehir Osmangazi University Faculty of Medicine, Department of Biostatistics and Medical Informatics, Eskişehir, Turkey

**Keywords:** hyperparathyroidism, Platelet function, P selectin, calcium, Bone loss

## Abstract

**Objective:**

Coagulation and fibrinolysis defects were reported in primary hyperparathyroid patients. However, there are not enough data regarding platelet functions in this group of patients. Our aim was to evaluate the platelet functions in primary and secondary hyperparathyroid patients and to compare them with healthy subjects.

**Materials and Methods:**

In our study 25 subjects with primary hyperparathyroidism (PHPT), 25 subjects with secondary hyperparathyroidism (SHPT), and 25 healthy controls were included. Platelet functions of the subjects were evaluated by using platelet-rich plasma and platelet aggregation tests induced with epinephrine, adenosine diphosphate (ADP), collagen, and ristocetin. Serum P selectin levels, which indicate platelet activation level, were measured in all subjects. Bone mineral densitometry was performed for all patients.

**Results:**

There was no significant difference between the groups with PHPT and SHPT and the control group regarding the platelet aggregation tests and serum P selectin levels. There was also no significant correlation between parathormone levels and aggregation parameters (ristocetin, epinephrine, collagen, and ADP: respectively p=0.446, 0.537, 0.346, and 0.302) and between P selectin (p=0.516) levels. When we separated the patients according to serum calcium levels, there was also no significant difference between aggregation parameters and serum P selectin levels between the patients with hypercalcemia and the patients with normocalcemia. We could not find any significant correlation between aggregation parameters, P selectin levels, and serum calcium levels in this group of patients. Bone loss was greater in patients with PHPT.

**Conclusion:**

There is no significant effect of PHPT or SHPT and serum calcium levels on platelet functions when evaluated by aggregation tests.

## Abstract (tr)

**Amaç:**

Koagülasyon ve fibrinoliz bozuklukları primer hiperparatiroidili hastalarda rapor edilmekle beraber bu hasta grubunda trombosit işlevlerine ilişkin yeterli veri yoktur. Bu nedenle primer ve sekonder hiperparatiroidisi olan hastalarda ve sağlıklı kontrol grubunda trombosit fonksiyonlarını değerlendirmeyi ve gruplar arasında farkı karşılaştırmayı amaçladık.

**Gereç ve Yöntemler:**

Çalışmamıza 25 primer hiperparatiroidisi (PHPT) olan hasta, 25 sekonder hiperparatiroidisi (SHPT) olan hasta ve 25 kontrol grubu dahil edildi. Trombosit fonksiyonları trombositten zengin plazma ve epinefrin, adenozin difosfat (ADP), kollajen ve ristosetinle trombosit agregasyon testleri yapılarak değerlendirildi. Trombosit aktivasyon düzeyini gösteren serum P selektin düzeyleri tüm hastalarda ölçüldü. Kemik mineral dansitometresi tüm hastalarda değerlendirildi.

**Bulgular:**

PHPT ve SHPT’li hastalar ve kontrol grubunun trombosit fonksiyon testleri ve serum P selektin düzeyleri arasında istatistiksel açıdan anlamlı bir fark saptanmadı. Parathormon düzeyi ile agregasyon parametreleri (ristosetin, epinefrin, kollajen, ve ADP: sırasıyla p=0,446, 0,537, 0,346 ve 0,302) ve P selektin (p=0,516) düzeyi arasında da anlamlı bir korelasyon saptanmadı. Hastalar kalsiyum düzeylerine göre hiperkalsemik ve normokalsemik olarak ayrıldıklarında da agregasyon parametreleri ve P selektin düzeyleri arasında anlamlı fark saptanmadı. Hasta gruplarımızda trombosit fonksiyonları, P selektin düzeyi, serum kalsiyum düzeyileri arasında istatistiksel açıdan anlamlı fark bulunmadı. Kemik kaybı PHPT’li olan grupta daha belirgindi.

**Sonuç:**

Agregasyon testleri ile değerlendirildiğinde PHPT veya SHPT ve serum kalsiyum düzeylerinin trombosit fonksiyonları üzerine belirgin etkisi yoktur.

## INTRODUCTION

Hemostasis is regulated by a balance between stimulators and inhibitors of platelet functions. The deterioration of the balance between inhibitors and stimulators of platelet functions results in thrombosis or bleeding. Platelets are involved in primary hemostasis, which includes the formation of a plug by the adhesion and activation of platelets in response to vascular damage or the loss of integrity of the vascular wall. Many physiological stimuli can activate platelets both in vivo and in vitro, such as collagen, proteolytic enzymes, and low-molecular-weight compounds. Clinically platelet functions are evaluated by platelet aggregation and activation tests [1,2]. The use of platelet agonists such as collagen, adenosine diphosphate (ADP), epinephrine, and ristocetin triggers classical platelet response and a great deal of information can be obtained from platelet aggregation. P selectin is a membrane glycoprotein within platelets and endothelial cells that is mobilized to the plasma membrane following cell activation and it is used to evaluate platelet activation [3,4].

It is well known that primary hyperparathyroidism (PHPT) is associated with a high risk of cardiovascular disease and increased mortality and morbidity related to cardiovascular problems [5,6,7]. There are also studies that relate hyperparathyroidism with a potential tendency toward hypercoagulation [8,9]. There are some cases of thrombotic events seen in the course of hyperparathyroidism [10]. However, knowledge about the effects of hyperparathyroidism on platelet functions is unsatisfactory and conflicting. Whereas elevated parathormone (PTH) levels and hypercalcemia are significant features of PHPT, PTH elevation does not accompany hypercalcemia in secondary hyperparathyroidism (SHPT). There are studies investigating the effect of serum calcium levels on platelet aggregation, coagulation, and thromboelastography in healthy people [11]. However, it is not clear whether hyperparathyroidism disturbs platelet function and if so whether it is related to the high PTH levels per se or to the accompanying hypercalcemia.

In this study we aimed to evaluate platelet functions in patients with both PHPT and SHPT.

## MATERIALS AND METHODS

Twenty-five subjects with PHPT, 25 subjects with SHPT, and 25 healthy age-matched control subjects were included in the study. The diagnosis of PHPT was based on clinical assessment and laboratory findings. Parathyroid adenomas were shown in all of the PHPT patients on both parathyroid ultrasound and ^99m^technetium scans of the parathyroids. Elevated PTH levels in the case of normal or low serum calcium level, vitamin D deficiency, and decreased urinary calcium excretion were regarded as signs of SHPT. Twenty-five healthy age- and sex-matched subjects with normal values of biochemical parameters were used as controls.

The purpose and the procedure of the tests were explained to the subjects and written informed consent was obtained from each participant. The experimental protocol was designed and performed according to the principles of the Declaration of Helsinki and it was approved by the Ethics Committee of the Eskişehir Osmangazi University Medical Faculty.

Serum calcium, phosphorus, albumin, chloride, and creatinine levels were measured for each of the subjects. Serum intact PTH was measured from venous blood samples at a central laboratory using a solid-phase two-site chemiluminescent enzyme-labeled immunometric assay with a reference range of 15-65 pg/mL. Serum calcium, phosphorus, and creatinine levels were measured colorimetrically. Serum albumin levels were measured by immunoturbidimetric assay and serum creatinine levels were measured by using an ion-selective electrode. Twenty-four hour urine collections were used in order to calculate urinary calcium excretion rates. Creatinine clearance (Ccr) levels were calculated according to the Cockroft-Gault formula. Patients with serum creatinine level above 1.2 mg/dL or Ccr level below 70 mL/min were not included in the study in order to exclude the confounding effects of renal failure on platelet functions. Tubular reabsorption of phosphate was calculated as TRP=[1-(up/pp)x(pcr/ucr)]x100.

Platelet functions of the subjects were evaluated by using platelet-rich plasma and platelet aggregation tests with epinephrine, ADP, collagen, and ristocetin. Serum P selectin levels, which indicate platelet activation level, were also measured in all subjects. Groups were matched with respect to age. Exclusion criteria included patients with known bleeding or other systemic disorders such as hepatic and endocrine diseases, acute infections, autoimmune disorders, or cancer, and a platelet count of less than 150x109/L or more than 450x109/L and a hemoglobin level of less than 10 g/dL. The patients did not receive agents that could affect platelet functions such as acetylsalicylic acid, ticlopidine, dipyridamole, or nonsteroidal antiinflammatory drugs in the 10 days prior to the platelet aggregation studies.

### Sample Collection and Laboratory Methods

Citrated blood was collected under light tourniquet through 19-gauge needles into 4.5-mL vacutainers (Becton Dickinson, USA) containing 3.2% trisodium citrate in a 9:1 blood/anticoagulant ratio. The collection was performed early in the morning after overnight fasting. Samples for blood counts were drawn into Becton Dickinson anticoagulated tubes and complete counts were made with a Beckman Coulter Gen-S SM (USA) automated blood counting device. Coagulation tests were performed with an ACL TOP Coagulation Analyzer (Instrumentation Laboratory, USA). Prothrombin time (PT) was measured with a HemosIL RecombiPlasTin kit (Instrumentation Laboratory), activated partial thromboplastin time (aPTT) was measured with a HemosIL SynthASil kit (Instrumentation Laboratory), and fibrinogen was measured with a HemosIL Fibrinogen-C XL kit (Instrumentation Laboratory). The normal ranges for these tests in our laboratory are: aPTT, 24-36 s; PT, 8-13 s; and fibrinogen, 200-400 mg/dL.

Platelet aggregation studies were performed with a whole blood lumi-aggregometer (Model 540-Ca, Chrono-log Corporation, USA) using an optical method according to the manufacturer’s instructions. Whole-blood specimens were centrifuged for 10 min at 200xg to obtain platelet-rich plasma. Platelet-poor plasma was obtained from the remaining specimens by recentrifugation at 200xg for 15 min. A platelet count was performed on the platelet-rich plasma and was adjusted to 300x103/µL with platelet-poor plasma. Next, 450 µL of this platelet-rich plasma was transferred into cuvettes (Chrono-log No: P/N 312), each containing a disposable siliconized bar. After agonist addition, platelet aggregation was measured over 6 min and expressed as a percentage of the maximal amplitude in platelet-rich plasma. The agonists used and their final concentrations were: ADP (Chrono Par 384), 5 µM; collagen (Chrono Par 385), 2 µg/mL; ristocetin (Chrono Par 396), 1.25 mg/mL; and epinephrine (Chrono Par 393), 5 µM. A commercially available ELISA method was used to determine serum P selectin levels (BBE6 catalog number, R&D Systems, USA). All analyses were performed in duplicate, and the mean value was used for statistical calculations. The levels of osteocalcin (2-22 ng/mL) and deoxypyridinoline (2.3-5.4 nM DPD/mM creatine) were measured. The bone mineral densitometry of the patients was studied and T scores were evaluated.

All statistical analysis was performed using SPSS 15 and SigmaStat 3.5. The distibution of variables was checked initially by Shapiro-Wilk test. Parametric tests were applied to data having normal distribution. Comparisons between 2 different groups were assessed by independent t-test and changes of variables within groups were assessed by paired samples t-test. Pearson correlation analysis was used to evaluate the relationships between variables. P<0.05 was accepted as indicating statistical significance. Results are given as mean ± SD.

## RESULTS

Basic characteristics of the study population are shown in Table 1. PTH levels of the patients with primary and SHPT were significantly higher than those of the control group (p<0.01). Serum calcium levels of the patients with PHPT were higher than those of both the patients with SHPT and the control group, as expected (p<0.001). There was no significant difference between hematological parameters such as hemoglobin, leukocyte and platelet counts, and PT levels among the groups. PTT and D-dimer levels were higher in patients with SHPT (Tables 1 and 2).

Patients with primary and SHPT are compared in Tables 1 and 3. PTH, serum calcium, urinary calcium excretion, and chloride/phosphorus ratios were higher in patients with PHPT when compared with SHPT (p<0.001). Tubular phosphate levels were low in patients with PHPT (p<0.001). When bone mineral densitometries were evaluated, femur neck bone density was lower in patients with PHPT (p<0.05). Osteocalcin levels were higher in patients with PHPT (p<0.001).

Platelet functions of the patients with primary and SHPT and the control group are shown in Table 2. Platelet functions evaluated by platelet aggregation induced by epinephrine, ADP, collagen, and ristocetin were not statistically different from each other. There was also no significant difference of P selectin levels among the groups. There was no significant correlation between either PTH or P selectin levels and platelet aggregation parameters.

Patients with primary and SHPT were divided into two groups according to serum calcium levels (Table 4). The first group included the patients with serum calcium levels equal to or higher than 10.5 mg/dL and the second group included the patients with serum calcium levels lower than 10.5 mg/dL. There was no significant difference between the two groups in respect to platelet aggregation studies induced by epinephrine, ADP, collagen, and ristocetin. P selectin levels also did not differ significantly between the groups. We could not find any significant correlation between aggregation parameters, P selectin levels, and serum calcium levels in this group of patients. Statistical values did not differ when serum calcium corrected for serum albumin level was used.

## DISCUSSION

Recent studies suggest that hyperparathyroidism has many systemic effects other than those on bone and mineral metabolism. PTH excess is strongly associated with prevalent and incident cardiovascular risk factors such as hypertension, diabetes, and cardiovascular diseases. There is also evidence connecting adverse cardiovascular outcomes, including death and incident coronary artery disease and myocardial infarction, to PTH excess [6,12,13,14]. Two biochemical features of hyperparathyroidism, namely elevated PTH levels and elevated serum calcium levels, may be implicated with those adverse outcomes. Although there are some studies suggesting that severe PHPT could impair vascular compliance and PTH rather than serum calcium levels being the casual factor, it is still uncertain which of the parameters is the main offending mediator in those circumstances [15].

Abnormalities in coagulation and fibrinolysis pathways have also been detected in PHPT, mostly supported by small case-control studies, and the evidence is still conflicting [8,9]. There are some case reports of thrombotic events associated with PHPT in which high serum calcium is accused of being a causative factor. In those cases, renal vein thrombosis and dermal necrosis due to thrombosis were encountered during the course of hyperparathyroidism [10,16,17]. Thrombotic events were reported also in SHPT [17]. The high incidence of vascular thrombosis seen in patients with hyperparathyroidism may represent a potential for hypercoagulation and may explain the increased cardiovascular morbidity in those patients.

In an early study on this topic, bovine PTH was shown in vitro to inhibit platelet aggregation and activation strongly [18]. Later, however, another study showed that platelet functions were not affected by synthetically manufactured PTH. The irregular platelet functions in the previous study were attributed by the authors to the additives used during the preparation of the bovine PTH [19].

In symptomatic primary hyperparathyroid patients, significantly higher plasma levels of tissue plasminogen activator and lower platelet activator inhibitor-1 (PAI-1) and tissue factor pathway inhibitor F levels compared to controls matched for age, sex, and body mass index were reported. Elevated PAI-1 levels found in patients with PHPT were proposed to be the causative factor for the tendency to thromboembolic events by lowering fibrinolytic activity. Those findings were suggested to represent a potential hypercoagulable and hypofibrinolytic state [9]. Increased platelet count, higher activities of factor VII and IX, and increased levels of D-dimer were also found in PHPT patients compared to healthy controls [8]. In another study, a positive relationship was found between PTH and PAI-1 levels in patients with PHPT without manifest cardiovascular disease [20]. However, hemostatic and fibrinolytic disorders of hyperparathyroidism are very rarely studied fields of research in the literature and there are not enough data on this subject. Platelet functions induced by ristocetin, ADP, collagen, and epinephrine were not studied in hyperparathyroid patients before and there is no study to date evaluating P selectin levels in hyperparathyroid patients. Moreover, all the studies evaluating the fibrinolysis and coagulation cascades were performed in patients with PHPT and do not indicate whether the elevated PTH levels or the high calcium levels were responsible for the results.

In our study, we could not find any significant differences among groups regarding platelet activation and aggregation studies. There was no significant correlation between PTH levels and aggregation parameters or serum P selectin levels. According to these results we concluded that primary and SHPT did not notably affect platelet functions. In this respect, this is the first study to show platelet aggregation and activation levels in both primary and SHPT. Contrary to the mentioned studies, D-dimer levels were higher in patients with SHPT, which make us think that high levels of PTH may cause a trend toward thrombosis independent of calcium levels. Another result of our study shows that lumbar and femoral bone loss was more pronounced in patients with PHPT. According to other studies bone mineral densitometry is decreased in hyperparathyroidism, and after parathyroidectomy bone mineral densitometry improves [21].

However, platelet functions could be affected by the levels of serum calcium of the patients independently of PTH levels. Therefore, we also evaluated the patients by separating the patients according to their serum calcium levels and compared the platelet functions of the patients with high serum calcium levels (≥10.5 mg/dL) with the patients with normal serum calcium levels (<10.5 mg/dL). Hypercalcemia almost always accompanies PHPT, but patients with SHPT are usually normocalcemic. Calcium levels are known to play a key role in the regulation of platelet functions. In a previous study, the effects of extracellular calcium concentrations on platelet aggregation, coagulation, and thromboelastography were studied in vitro in blood samples collected from healthy subjects [11]. In that study it was shown that high calcium levels could inhibit platelet aggregation, coagulation factor activity, and blood coagulation; the level of calcium found to affect platelet functions was ≥15 mg/dL [11]. In our study, we could not show any significant difference regarding platelet aggregation studies and serum P selectin levels between the patients with high and normal serum calcium levels. We concluded that serum calcium levels did not significantly alter platelet functions. However, it is possible that our findings might be related to the fact that our patients’ average calcium levels were not as high as in the previous study. In our patient group the highest serum calcium level was 12.4 mg/dL, and when corrected according to serum albumin level, this reached 13.9 mg/dL at most. Moreover, in the previous study, in vitro calcium levels were used. In another study mean platelet volume was used to evaluate thrombocyte activation in patients with PHPT and platelet activation was found to be increased [22]. However, mean platelet volume is not a valuable measure for platelet activation.

In conclusion, in this study we showed that platelet aggregation did not change in either primary or SHPT. However, since we did not study platelet aggregation inhibition, we cannot say clearly with the existing data whether there is a tendency toward thrombosis or not in hyperparathyroidism.

## Ethics

Ethics Committee Approval: Eskişehir Osmangazi University Ethics Committee 29 May 2009 (approval number: 11); Informed Consent: It was taken.

## Figures and Tables

**Table 1:**
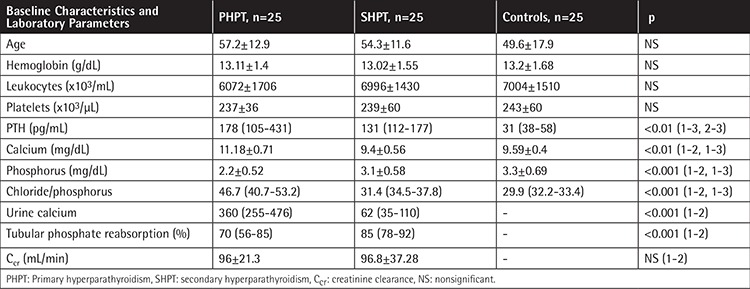
Baseline characteristics and serum laboratory parameters of the study population.

**Table 2:**
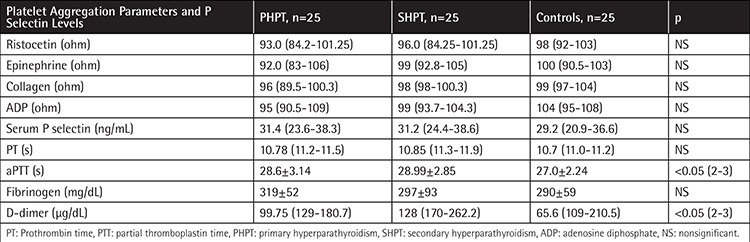
Platelet aggregation studies and P selectin levels of the patients and the control group.

**Table 3:**
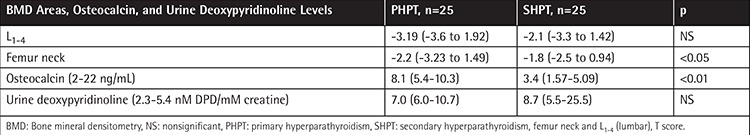
Comparison of bone mineral densitometry values, osteocalcin, and urine deoxypyridinoline levels of patients with primary hyperparathyroidism and secondary hyperparathyroidism.

**Table 4:**
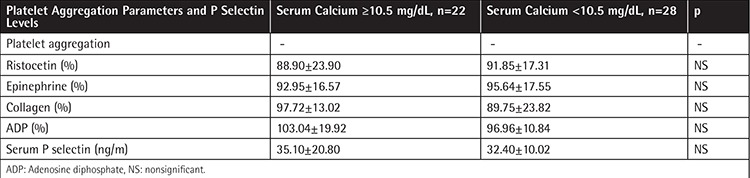
Platelet functions of the patients classified according to serum calcium levels.
